# The “Primitive Brain Dysfunction” Theory of Autism: The Superior Colliculus Role

**DOI:** 10.3389/fnint.2022.797391

**Published:** 2022-05-31

**Authors:** Rubin Jure

**Affiliations:** Centro Privado de Neurología y Neuropsicología Infanto Juvenil WERNICKE, Córdoba, Argentina

**Keywords:** autism spectrum disorders, superior colliculus (SC), neurodevelopmental disorders, brainstem, early brain development, autism pathogenesis, autism theories

## Abstract

A better understanding of the pathogenesis of autism will help clarify our conception of the complexity of normal brain development. The crucial deficit may lie in the postnatal changes that vision produces in the brainstem nuclei during early life. The superior colliculus is the primary brainstem visual center. Although difficult to examine in humans with present techniques, it is known to support behaviors essential for every vertebrate to survive, such as the ability to pay attention to relevant stimuli and to produce automatic motor responses based on sensory input. From birth to death, it acts as a brain sentinel that influences basic aspects of our behavior. It is the main brainstem hub that lies between the environment and the rest of the higher neural system, making continuous, implicit decisions about where to direct our attention. The conserved cortex-like organization of the superior colliculus in all vertebrates allows the early appearance of primitive emotionally-related behaviors essential for survival. It contains first-line specialized neurons enabling the detection and tracking of faces and movements from birth. During development, it also sends the appropriate impulses to help shape brain areas necessary for social-communicative abilities. These abilities require the analysis of numerous variables, such as the simultaneous evaluation of incoming information sustained by separate brain networks (visual, auditory and sensory-motor, social, emotional, etc.), and predictive capabilities which compare present events to previous experiences and possible responses. These critical aspects of decision-making allow us to evaluate the impact that our response or behavior may provoke in others. The purpose of this review is to show that several enigmas about the complexity of autism might be explained by disruptions of collicular and brainstem functions. The results of two separate lines of investigation: 1. the cognitive, etiologic, and pathogenic aspects of autism on one hand, and two. the functional anatomy of the colliculus on the other, are considered in order to bridge the gap between basic brain science and clinical studies and to promote future research in this unexplored area.

## Introduction

Among the cardinal symptoms of autism spectrum disorder (ASD) are an inability to properly direct gaze and abnormalities in attention to appropriate targets. The superior colliculus (SC) is a brainstem structure with sentinel functions (Merker, 1980), which is also tasked with the ability to redirect both gaze and attention. It also activates emotional and motor networks to produce responses congruent with the stimuli. To do this, it has widespread connections with structures located throughout the brain. In this article, we set forth the reasoning underlying a new model that places early compromise of collicular function as a central feature of ASD, not only because of these gaze and attention symptoms but also because an early loss of these collicular capabilities leads to improper development of numerous other systems, explaining not only the language and social skill deficits but the full ASD clinical syndrome.

Regardless of their etiology, all neuropsychiatric disorders are syndromes exclusively defined by their clinical manifestations: a cluster of symptoms determined by the compromise of specific functions or neural systems. For example, several etiologies (cerebral infarct, abscess, etc.) affecting specific language networks will result in Broca's aphasia. In two different individuals with the same syndrome, the severity of the symptoms can be very variable, but the more similar their clinical manifestations are, the more similar their pathogenesis will be.

Among all developmental disorders (DD), ASD (or autism) is the most complex because it compromises numerous functions—social, communicative, sensory/motor, emotional, attentional, autonomic, etc. (Tuchman, 2006; American Psychiatric Association, 2013; Eilam-Stock et al., 2014). For this reason, it seems unwise to look for a specific brain area or system responsible for the various clinical manifestations. However, what renders ASD highly coherent is that this heterogeneous cluster of symptoms is present in almost every individual with the syndrome, in spite of their cognitive level- from severe deficiency to high intellectual ability.

Autism spectrum disorder coherence is not explained by etiologic factors because several prenatal or postnatal genetic or non-genetic conditions could result in ASD (Jure, 2019; Martin et al., 2022). Similarly, several autism theories have been proposed, from dysfunctions of sensory, motor, autonomic, attentional, or emotional basic processing (for example abnormal social motivation) to exclusive higher-order compromise (theory of mind, social cognition, executive functions abnormalities, etc.) (see references below). The compromise of a brain hub underlying perceptual, emotional, motor, attentional or cognitive abilities could be the common factor responsible for the repetition of disparate symptoms in most ASD individuals. Additionally, the presence of early brain overgrowth (Courchesne et al., 2007) and abnormalities in the synaptic organization during the first months of life (Vértes and Bullmore, 2015), as well as the timing of clinical presentation circumscribe the search to networks with key innate functions impacting specific aspects of neurodevelopment, while sparing others. This could explain the uneven skills and the coexistence of ASD with high intelligence.

A central aspect of this article is the concept of *consciousness*. There is an intense debate regarding its definition and hence the neural substrate supporting it. The more generalized, corticocentered view, maintains that it is only limited to overt or explicit aspects of reflective self-consciousness, corresponding to an adult human perspective. Some feel that consciousness is *not* the sole preserve of the cerebral cortex. Penfield (1952) based this view on clinical and physiological observations in epilepsy, while more recently, Merker (2007) based his on animal studies and on behaviors of children born without a cerebral cortex. The latter author's proposal includes a continuum of consciousness; the lowest is sustained only by upper brainstem nuclei including the SC, and the highest by the mature human cortex. The former involves the implicit sense of one's own body and the environment in order to select a target and to act due to motivation or emotions. Different degrees of complexity were added during phylogenetic evolution due to the gradual expansion of the telencephalon. These more elaborated perceptual and cognitive characteristics of consciousness reached their highest levels to finally include self-consciousness in great apes and humans, along with the addition of language in the latter. The upper brainstem organization, common to all vertebrates, provides the functional properties not only to support wakefulness but to accomplish basic behaviors in order to survive. These functions dominate the early stages of human development and are central to the theory of mind (Watt, 2007) and to most ASD theories. Additionally, they continue to be essential during all life in order to process the surfeit of covert information provided to the individual and to react automatically whenever necessary (see Merker, 2007 for a thoughtful discussion on this topic). Accordingly, the terms *covert, implicit*, or *automatic* will be used in this article instead of *non-conscious* or *unconscious* attention or behavior.

In the present brain-based framework, previous ASD theories and etiologies will be included as pieces of a more complex puzzle. The objective is to analyze how disruptions of the SC might help explain the whole clinical syndrome and its paradoxes.

## Autism Theories

### The Role of Vision in ASD

Visual deficits are not included among ASD symptoms (American Psychiatric Association, 2013), and it is well-known that most ASD individuals excel at visual skills. Hence, it seems illogical to point to visual abnormalities as responsible for the syndrome. However, two main facts discussed below contradict this posture: the strong association of congenital blindness (CB) with ASD (Jure et al., 2016) and the fact that most ASD theories are based on the presence of abnormal visual attention and processing.

### CB and ASD

Unlike congenital deafness which severely compromises oral language, but is not associated with higher rates of ASD (Jure et al., 1991), total CB conveys a very high prevalence of ASD (Mukaddes et al., 2007; Jure et al., 2016; Kiani et al., 2019). A study comparing children with severe vs. profound congenital visual impairment showed that even the presence of very coarse vision of forms in the first condition (instead of a total lack of vision shown by the second) during the first months of life was a protective factor lowering the presence of developmental setback with autistic-like symptoms (Dale and Sonksen, 2002). *These findings might reflect the importance of very early visual input on social and communicative development*. Additionally, autistic symptoms in CB are not only limited to social and communicative deficits. Stereotypies and repetitive behaviors (Mink and Mandelbaum, 2006), as well as affective disorders (Wigham et al., 2017), are very frequent and very similar in both CB and ASD. Moreover, mood disorders are more common among children and young adults with early and severe visual impairment (Augestad, 2017).

(See Box 1 for anecdotal but relevant clinical examples of CB and ASD).

Box 1 **Clinical examples of congenital blindness and ASD.**

The present author examined a congenitally blind teenage girl with social and communication difficulties and abnormal prosody, but with excellent semantic and syntactic abilities, high level reasoning, and academic skills. Another example (by personal communication) is a social worker with CB who admitted identifying herself in some descriptions of the autistic profile given by a lecturer in a conference about autism. She was also fascinated by the notion *of facial blindness* because “I've been blind from birth and not only have I not seen a face but I have no concept of a face,” she added. Another example of the effect of total lack of form vision during the first year of life is the neurodevelopment of two identical twins I followed from infancy until adolescence (same genetic background and environmental conditions). The same etiology_:_ retinopathy of prematurity_:_ provoked total congenital blindness in one of them and partial blindness in the second, with preserved ability to see forms and movements. The first child displays all the myriad of ASD symptoms with severe mood disorders. The second child instead, is empathic, sociable, communicative, cares about his brother, and his mood is usually good and stable. These examples, although anecdotic, reveal the importance of early visual input on the pathogenesis of ASD.

### Abnormal Visual Attention in ASD

Long before the development of language and social cognition, early life visual attention to faces and movements is supported by a non-canonical, rapid subcortical visual system that accesses the amygdala by way of an SC-pulvinar pathway (Johnson, 2005; McCleery et al., 2007). This stream is predominantly magnocellular and has the ability to automatically detect movement or rapidly changing images. In contrast, the canonical visual route reaches the primary visual cortex *via* the lateral geniculate nucleus (LGN). Although it also receives magnocellular input in two layers, it is predominantly parvocellular, with four layers receiving inputs. While effectively integrated by rich interconnections, the magnocellular system simultaneously processes a great deal of global and dynamic information with a coarse grain. The parvocellular system processes mostly *static*, focused, detailed, and chromatic features of the environment (McCleery et al., 2007; Nassi and Callaway, 2009).

Several well-known theories of ASD point directly or indirectly to a predominance of the parvocellular over the magnocellular visual system***:*** *the Magnocellular Dysfunction Theory* (Milne et al., 2002; McCleery et al., 2007; Laycock et al., 2020), *the Weak Central Coherence (WCC) theory* (Frith and Happé, 1994), and *the Enhanced Perceptual Functioning (EPF) model* (Mottron and Burack, 2001). *The most consistent finding of ASD is an automatic initial attentional bias to local stimuli rather than global processing* (Happé and Frith, 2006; Guy et al., 2016). Typically, healthy individuals automatically perceive the whole picture before explicitly attending to details. A recent meta-analysis review strongly suggests that this pattern is reversed in ASD (Van der Hallen et al., 2015).

There is a weakness regarding diffuse magnocellular dysfunction as responsible for ASD. A generalized magnocellular dysfunction involving both, implicit/subcortical and explicit/cortical magnocellular networks, does not explain why a proportion of individuals with ASD have a normal explicit perception of biologic movements or facial clues and mainly fail in automatic processing (Sato et al., 2010). Additionally, exclusive dysfunction of visual magnocellular functions can explain some, but not all ASD symptoms (Happé and Frith, 2006).

### Abnormal Face Processing and Abnormal Social Motivation

Several studies have revealed the presence of abnormal face processing in individuals with ASD (Elgar and Campbell, 2001; Carver and Dawson, 2002; McCleery et al., 2007; Kleinhans et al., 2008; McPartland et al., 2011; Senju et al., 2011; Elsabbagh et al., 2012; Stavropoulos et al., 2018; Morgan and Hills, 2019; Safar et al., 2020; Shephard et al., 2020). *A consistent finding is the presence of abnormalities in the initial, covert perception of faces by subcortical structures* (Sato et al., 2010, 2016; Akechi et al., 2014; Antezana et al., 2016; Naumann et al., 2018; Bathelt et al., 2021). Strong innate behavioral bias neonates share with other vertebrates is the predisposition to follow faces and biological motion, and to pay attention to the gaze of others (Goren et al., 1975; Johnson et al., 1991; Rosa Salva et al., 2015; NIDA-Network et al., 2016). Besides the *innate attraction* to congeners, this process also requires the ability to *recognize* complex images, *decide implicitly* which one to select, and *integrate* internal states with information from a variety of sensory inputs (Chen and Hong, 2018) to finally *activate* premotor and motor commands in order to *perform* the action.

The *social motivation theory of ASD* proposes that dysfunctions of innate social motivational mechanisms affect the development of social cognition later in life. These behaviors are sustained by “experience-expectant”^1^ networks (Carver and Dawson, 2002; Chevallier et al., 2012) It has been suggested that developing this capability requires the interaction of several neurotransmitters (oxytocin, dopamine, and glutamate, as well as endogenous opioids) (see references in Chevallier et al., 2012).

The idea that subcortical structures supporting innate social motivation are compromised in ASD was questioned by follow-up studies of children *from 2 months* of age with a high family risk of ASD. These studies found normal visual attention for following faces in these infants (Johnson, 2014; Klin et al., 2015). However, another follow-up study *from birth* revealed that visual preferences for social stimuli strikingly differed between high-risk and low-risk newborns. The authors proposed a U-shaped curve in high risks infants: they started with abnormalities at birth, showed improvement when cortical structures start to mature and take control, and then suffered a regression due to the absence of a normally functioning subcortical mechanism to support the developing cortical areas (NIDA-Network et al., 2016). Prenatal exposure to valproic acid in humans frequently results in ASD (Bescoby-Chambers et al., 2001; Williams et al., 2001; Christianson et al., 2008). In a parallel animal model, it has been shown that visual social orienting mechanisms and social predisposition are selectively abolished in chicks by embryonic exposure to valproic acid (VPA) (Sgadò et al., 2018).

Early postnatal visual experience is necessary to produce the plastic changes required for normal facial processing (Carver and Dawson, 2002) and to shift from subcortical to cortical control (Morton and Johnson, 1991). Specific cortical regions, like the fusiform area normally employed for explicit face processing, may instead be devoted to other tasks depending on the experience and the expertise of the individual (e.g., bird identification or chess scene analysis) (Gauthier et al., 1999; Bilalić, 2016). Notably, activation of the fusiform gyrus and amygdala by cartoon characters, but not to faces, was observed in an autistic boy by fMRI (Grelotti et al., 2005).

### Abnormal Attentional ASD Theories

From the first descriptions of ASD to the present, many experts have maintained that early deficits in various attentional aspects have an exponential effect on development, resulting in the full clinical ASD syndrome (see Jure, 2019 and references therein). Early *orienting attention* to biological stimuli is critical for social development and it was found to be abnormal at 7 months in those at risk for ADS (Elison et al., 2013). While overt attention is essentially directed at a single target, cover attention analyzes several stimuli at once. A number of authors have also pointed to deficits in *divided attention during* complex events requiring the *simultaneous* analysis of various factors (Murray et al., 2005; Fletcher-Watson et al., 2008; Jaworski and Eigsti, 2015; Keehn and Joseph, 2016; Unruh et al., 2016; Bolis and Schilbach, 2017; Arora et al., 2022). Social events are the most demanding, as they require simultaneous attention to the internal state of the individual, to the environment, to the behaviors of others, and even to the hidden logic or real intentions behind such behaviors. Skorich et al. (2017) found a negative relationship between autism quotient and shared-attention, which is pivotal for constructing the self-other-object relationship.

Other authors have pointed to *abnormal attention disengagement* as the main factor responsible for ASD (Landry and Bryson, 2004; Elsabbagh et al., 2013; Keehn et al., 2013, 2021). Again, the superior colliculus plays a critical role in shifting attention to a new target.

### Abnormal Sensorimotor and Autonomic Processing in ASD

The abnormal response to sensory stimuli in individuals with ASD is highly variable. There is not a single pattern; multiple senses may be involved (auditory, visual, pain, temperature, taste, vestibular, olfactory, and somatosensory) and both hypo- and hypersensitivity have been described (see Rapin, 2006 for a detailed review). The following theories consider abnormal sensorimotor processing as responsible for ASD:

The *Intense World Theory* proposes excessive functioning of microcircuits and enhanced brain functioning as the underlying abnormality responsible for ASD. The animal model of autism used was also valproic acid exposed rat offspring. The authors considered that impaired habituation to sensory stimulation measured *in vivo* by the level of pre-pulse inhibition (PPI) was one of the main features of the hyper-reactivity and enhanced brain functioning in ASD individuals (Markram et al., 2008; Markram and Markram, 2010).

For more than five decades *Multisensory processing abnormalities* have been described in individuals with ASD (Camarata et al., 2020) and proposed as the abnormality responsible for the syndrome (Stevenson et al., 2014; Camarata et al., 2020; Kawakami et al., 2020; Siemann et al., 2020). More recently, a *Bayesian model of ASD* was proposed based on new computational frameworks suggesting that multisensory integration follows Bayesian rules of causal inference (French and DeAngelis, 2020). Palmer et al. (2017) hypothesize that autism is characterized by a greater weighting of sensory information in updating probabilistic representations of the environment. For the authors, this seminal abnormality results in abnormal actions, explaining full-blown ASD syndrome. Bayesian responses are only acquired after repeated exposure to similar events and require an interaction of perceptual, cognitive, and biologic mechanisms (Thaler et al., 2021). Consequently, this ability necessitates a bidirectional (both bottom-up and top-down) postnatal training process using first line, sentinel structures in order to automatically orient the attention to relevant stimuli and ignore non-relevant stimuli.

Integrative theories including motor aspects, such as *the micro-movement perspective theory*, maintain that ASD individuals have an early-life disruption of integration of sensory, motor, and autonomic aspects of the connections between the peripheral and the central nervous systems, which affects the stochastic rhythms of motions (e.g., speech gestures, eyes, facial micro-expressions, head, body, limbs, etc.). This compromises flexible transitions between intentional and spontaneous behaviors (Torres et al., 2013). The logical place for such disruption would be in the brainstem. Impaired acquisition of skilled motor acts, including *dyspraxia*, is frequently found in individuals with ASD, and abnormalities point mainly to a compromise of implicit learning with an excessive reliance on explicit/declarative learning (Gidley Larson et al., 2008). This finding might be indicative of cortically mediated learning, reflecting a more unifocal explicit learning without proper implicit/multifocal support. This may explain the frequent observation of uneven motor skills in this population.

The highest integration is required during dyadic interactions involving motor, emotional, cognitive, conversational, physiological, and neural aspects of *Interpersonal Synchrony*. Development of this capability starts at 2–3-month-old when the infant and mother nonverbally communicate by *intersubjective*, rapid, reciprocal, bidirectional visual-facial, auditory-prosodic, and tactile-gestural means (Schore, 2021). *Atypical interpersonal synchrony* from early life has also been proposed as an early marker of ASD (Koehne et al., 2016; McNaughton and Redcay, 2020).

### Mood Disorders and Alexithymia

There is no pattern of emotional profiles among individuals with ASD. They can vary from a significant lack of reaction to exaggerated behavioral outbursts, and from severe baseline mood disorders to a prominent lack of emotional expressions. What is confirmed is that individuals with ASD are at increased risk of suffering psychiatric conditions during their lifespan, with more prevalence of anxiety and mood disorders (see Rosen et al., 2018 for a review). A related psychiatric disorder, *Alexithymia*, is also highly prevalent among ASD individuals (Kinnaird et al., 2019; Morie et al., 2019). This disorder presents with difficulties in recognizing and expressing a variety of emotions and body sensations, a lack of imagination or fantasy life, and a tendency to focus on external, rather than internal, experiences (Sifneos, 1973). Links between cognitive function, body sensations, affective dimension, alexithymia, empathy, and ASD have been established by several authors (Grynberg et al., 2010; Murphy et al., 2017; Mul et al., 2018). Interestingly, autistic adults have recently described their own interoceptive difficulties as limited awareness of hunger, satiation, or thirst, as well as abnormal awareness or understanding of affective arousal, pain, or illness, and difficulty differentiating benign body signals from signals that represent medical concerns (Trevisan et al., 2021). These symptoms point to very basic, as opposed to higher-order, dysfunction or compromise in the loops that integrate brainstem structures with higher brain areas.

### Autonomic and Circadian Dysfunction

Basic neurological functions such as abnormalities in autonomic processing (Hirstein et al., 2001; Eilam-Stock et al., 2014) and circadian dysfunctions (Lorsung et al., 2021) are frequently found in ASD individuals, and these have also been pointed to as responsible for disruptions of emotional/social development. The cluster of abnormalities in autonomic/arousal regulation, sleep-wake homeostasis, and sensorimotor integration during the first months of life, *reflect brainstem involvement* and have been proposed as a very early risk factor for ASD (Burstein and Geva, 2021).

### Clinical Paradoxes and IQ Variability

None of the previous theories taken in isolation can explain the myriad of ASD symptoms and they do not clarify some frequent clinical paradoxes. For example, some individuals with confirmed ASD display excellent sports skills or write imaginative tales (author's clinical observations). Additionally, there are several examples of famous athletes, actors, or political leaders with suspected or confirmed ASD (ONGIG, n.d.). While anecdotal, these examples show that at least in some circumstances, sensory/motor integrative skills and/or mental abilities are either spared, or they are different, but not severely compromised. While some would argue that these are just idiosyncratic examples, their presence argues the ASD is not inherently a disorder of higher cerebral functions. In fact, ASD individuals can excel at *any* skill, depending on both, the pattern of their higher-order network losses, and the narrow range of interest the subject displays during their development. The result fosters “hypertrophy” in particular skills to the detriment of others. In fact, well-developed rational and logical abilities and the presence of high IQ levels in some individuals with ASD prompted the theory proposing *Autism as a disorder of high intelligence* (Crespi, 2016). The fact that the presence of superior cognitive skills does not preclude the expression of the full clinical syndrome points to a compromise of more primitive, instead of higher-order cortical networks.

A common factor that is *always abnormal* in ASD is the lack of appropriate development of *primitive behaviors* necessary to respond appropriately depending on the context. These manifestations are not always severe, such as a complete lack of social interest or communication. However, even mildly affected, high functioning, autistic individuals always display at least subtle symptoms, and these can have potentially severe consequences in their lives. It is well-known that they can exhibit a great variety of symptoms, from total lack of fear of strangers or real dangers to generalized fear or severe social anxiety. Mildly affected children will display an innate interest in interacting with others, but they will often do it inappropriately. For example, during a first meeting, they will try to hug, touch, or push the unfamiliar individual. They may not react at all to an aggressor or they might react in an exaggerated fashion to a mild innocent joke. Some do not understand the concept of authority and can treat adults like their peers, but at the same time, they do not know how to play with other children. Although these behaviors also involve higher-order mental abilities, they are universally present in normal children and animals. The latter must differentiate familiar from strange congeners, respect hierarchies, defend themselves and play with their peers in order to develop survival skills from a very early age. This suggests these behaviors are widespread in animals, and so must be part of the normal development of circuitry patterns.

### Neurodevelopmental Courses

Another consistent finding of ASD is the timing of onset of clinical manifestations. It occurs before 3 years of age. The usual initial manifestation is a delay or deviation in the emergence of normal social and communicative milestones from the beginning. A significant proportion will show, mostly between 18 and 24 months of life, a pattern of autism *regression*, where they lose early acquired language, social and communicative abilities (Rapin and Tuchman, 2006; Ozonoff and Iosif, 2019). After this initial period of regression, the rest of the evolution is similar to the other DD: a chronic *static* encephalopathy. Most ASD individuals will *partially* improve over time, but their core issues with social and communicative abilities will *remain life-long* and show varying degrees of compromise. A small proportion with *optimal outcomes* (3–20%) will improve significantly, developing acceptable communicative and social abilities in adulthood (Fein et al., 2013). However, even these adults will not enjoy non-predictable or highly demanding social environments that require appropriate spontaneous reactions in quality and timing. *These diverse developmental courses might reflect different pathogenic mechanisms as a result of either, primary abnormalities in subcortical structures or abnormal interactions of bridge networks between cortical and subcortical structures*.

*It is worth noting that all previous developmental courses are replicated in individuals with CB indicating that the absence of vision from birth might share similar pathogenic effects on the developing brain compared with other ASD etiologies* (Jure et al., 2016).

### Conclusions About ASD Theories and Clinical Manifestations

Autism theories are almost as diverse as autism symptoms. Almost every aspect (perceptive, attentional, autonomic, motor, integrative, emotional, etc.) has been pointed to as the seminal compromise responsible for the full clinical syndrome. This speaks to the significant interdependence between them. Complex behaviors, including socialization and communication, require a highly dynamic balance between these variables, and the dysfunction of one of them will affect the rest. Nevertheless, it is certainly possible that the clustering of ASD symptoms is just an epiphenomenon of a compromise of a brain hub supporting all these functions, as opposed to a cause-effect relationship between them.

*We can conclude that the most common aspects of ASD theories are the compromise of implicit mental processes, such as the initial bias in attention to biological events (faces, movements, etc.) or to global instead of local processing, as well as deficits in the automatic integration of simultaneous sensory, emotional, cognitive and motor dimensions*. The dysfunction of brainstem structures, which are normally mature at birth may produce pivotal disorders of input-processing-output behaviors, and so might explain several aspects: the full clinical syndrome, the early life manifestations, and the preservation of higher-order cerebral functions resulting in uneven skills.

## The Brainstem And The Sc

### The Primitive Subcortical Brain vs. the Cortical Brain

Every behavior is a motor act requiring the integration of different senses, mostly led by vision, with emotional needs. Brainstem nuclei and loops control the input-processing-output machinery in order to produce primitive behaviors without the involvement of higher neural networks. Among the several subtelencephalic nuclei receiving retinal input, the essential structure to accomplish this process is the tectum, called superior colliculus in mammals (Sewards and Sewards, 2002). Its preservation from the earliest vertebrate to humans bespeaks its irreplaceable role in adaptive behaviors (Basso et al., 2021). From a simple tri-synaptic loop, used to display avoidance or escape responses to looming images in lampreys, its complexity significantly increased during evolution. Along with changes in its cortex-like organization, several collicular-brainstem nuclei loops mediating diverse emotional, autonomic, and hormonal functions developed during phylogenesis (see Isa et al., 2021 for a review on this topic).

Besides the downstream outputs to premotor areas for very fast visuomotor transmission, the SC sends connections to the pulvinar, and other thalamic nuclei, which in turn provide access to the amygdala, striatum, and cerebral cortex. This *retino-colliculo-thalamic* pathway indirectly and simultaneously activates several *visual and non-visual* cortical regions. In turn, the SC receives massive *direct* cortical feedback.

The main visual route in humans is the *retino-geniculate pathway, which transmits* 90% of the retinal input. The dorsal lateral geniculate nucleus (dLGN) is the main nucleus responsible for central, foveal retinal vision. It is devoted to an explicit, detailed, and colorful analysis of the environment. Unlike the SC, the dLGN is *exclusively* visual and only directly reaches the striate visual cortex. Hence, in order to produce a behavior, a *slow* sequential process is initiated to integrate vision with other non-visual cortical regions, evaluate possible actions, make an overtly conscious decision, and finally send a motor command from frontal regions. Although these pathways are highly interconnected and we perceive vision as a unitary process, the retino-colliculo-thalamic pathway mediates covert visuo-motor behaviors, while the retino-geniculate pathway is devoted to detailed overt conscious processing (Isa et al., 2021). There is strong evidence that the human retino-colliculo-thalamic pathway develops much earlier than the retino-geniculo-cortical pathway, and the latter is probably not fully functional until nearly 2 months after birth (Sewards and Sewards, 2002; Bridge et al., 2015).

### Emotions and Brainstem Structures

Emotions have a strong and pervasive influence on human behavior as a whole (Schore, 1994; Lerner et al., 2015; Hogeveen et al., 2016; Keltner, 2019), but as we have noted, are not appropriately tied to behavior in ASD. *Primitive emotions* are innate and universal, and they determine orienting biases to environmental phenomena. They also modulate sensory experience, arousal, and autonomic functions, but their ultimate goal is to initiate actions that promote survival in animals and humans (Damasio, 1996; Venkatraman et al., 2017). Being genetically determined, it is not necessary to teach a child a primary emotion like fear, anxiety, anger, joy, or panic (Davis and Montag, 2019). On the other hand, feelings cannot be taught; for example, when it is not innately present, it is a big challenge to teach a child the *motivation* for socialization, communication, or play.

Recent studies in animals and humans disclose that the nuclei and networks which convey primary emotional systems are located in the brainstem (Panksepp and Biven, 2012; Venkatraman et al., 2017; Davis and Montag, 2019). On the other hand, higher cortical networks, which regulate emotions, are *experience-dependent* blank slates at birth that need to be trained by primitive structures and environmental influence (Schore, 1994). Ample evidence in animals and humans that primary emotions do not require the neocortex is reviewed by Davis and Montag (2019). Even primitive structures like the amygdala are dependent on the input of brainstem nuclei. For example, selective ablation of the periaqueductal gray matter (PAG), but not of the amygdala, abolished rage responses in animals (Bailey and Davis, 1942, 1944). Similarly, exclusive ablation of the SC provoked a total absence of fear of snakes in monkeys (Soares et al., 2017).

The self-sustained and holistic process of brainstem functions explains why neonatally decorticated rats still displayed complex survival/emotional behaviors (Siviy and Panksepp, 1985). These rats were able to reproduce and care for their pups. They also exhibited the same ability to play as control rats and to display aggressive, defensive, or *conditioned* (learned) freezing behaviors. In contrast, rats with small lesions in the parafascicular region of the thalamus significantly reduced play time, motivation, and play solicitation. Evidence in humans with congenital or acquired cerebral lesions also points to subcortical structures acting as substrates for primary emotions (Merker, 2007; Damasio et al., 2013).

### The Superior Colliculus

The SC is prepared to perceive relevant external events, mainly biological ones before they reach overt consciousness. The use of canonical pathways from external receptors of each specific sensory modality and then to association and motor cortical areas to accomplish this would be very slow and therefore less useful. The SC superficial layers are exclusively visual and receive direct retinal input from magnocellular and koniocellular neurons (May, 2006; Basso et al., 2021). This visual information is integrated with auditory, somatosensory, and vestibular input within the intermediate/deep layers. In order to react *automatically*, the SC has not only the ability to perceive the stimulus but also to make an implicit decision and finally send the corresponding motor command (Gandhi and Katnani, 2011; Basso and May, 2017; Farrow et al., 2019; Basso et al., 2021). By accurate saccades, it orientates central vision to objects of interest. Then, the canonical visual pathway is activated allowing a detailed analysis of the event. None of the other brain hubs seem to have the key strategic location to function from birth as a first-line monitor and first reactive system (Soares et al., 2017).

At present, it is clear that the SC is not simply a structure for producing reflex movements. It has rich connections with the forebrain through ascending networks and is also well-connected with numerous brainstem nuclei. There is compelling evidence of its role in cognitive, attentional, emotional, and complex, higher-order behaviors (May, 2006; Basso and May, 2017; Basso et al., 2021). This aspect and the fact that it is ready to function at birth as an experience-independent structure is a strong argument in favor of its possible role in neural development. As was previously remarked, late maturing, experience-dependent cortical structures require the information received during the first months of life. Hence, any small abnormality or deviation in SC functions could have significant consequences on neurodevelopment.

### The SC Is Responsible for the Initial Global Vision and Facial Detection

Against the traditional view that SC functions are limited to output processes of gaze control, selective attention, and target selection properties, there is evidence for its having an intrinsic *visual perceptive role* in gestalt, coarse rapid object detection, and object identification, as well as in analyzing luminance contrast and motion stimuli (Sewards and Sewards, 2002; Schneider, 2005; Georgy et al., 2016; Chen and Hafed, 2018).

While the pulvinar (Pul) and the LGN receive input from the magnocellular and parvocellular visual system, there is evidence that the SC/Pul pathway is responsible for the automatic *bias* toward magnocellular global processing (Lomber, 2002; Tamietto, 2011; Sato et al., 2016; Petry and Bickford, 2019; Laycock et al., 2020). It has been shown in animals that selective inactivation of the visual superficial layers of the SC during *pattern* discrimination learning reverses the precedence for global visual features that is typical of normal learning (Lomber, 2002).

Findings in favor of the SC/Pul role in automatic alerting or orienting responses are the presence of a *direct subcortical* SC/Pul pathway to the amygdala (Amy) (see above) (Laycock et al., 2020), and the fact that the SC simultaneously triggers visual and *non-visual* sensory and motor cortical structures, allowing a reaction before the stimulus reaches conscious explicit appreciation (Petry and Bickford, 2019). Instead, the information transmitted by the LGN travels first through a series of visual cortical areas, before accessing the Amy, which in turn influences non-visual sensory and motor cortical structures. Most evidence in humans indicates that attentional selection for emotional stimuli is under bottom-up control, even in adults, and is mediated by the SC/Pul/Amy circuits activated by the magnocellular system (Vuilleumier et al., 2003; Vuilleumier, 2015; Mulckhuyse, 2018; McFadyen, 2019; Laycock et al., 2020; McFadyen et al., 2020). Interaction of several subcortical structures plays a key role in multiple aspects of normal face perception during life (Gabay et al., 2014) and it has been demonstrated that the SC, the pulvinar, and the amygdala all support evaluation of facial traits in blindsight (Kinoshita et al., 2019; Ajina et al., 2020). During social interactions, the recording of multiple subtle cues from emotional expressions that depend on the fleeting eye, mouth, face, and body movements are also processed by magno/koniocellular networks (Laycock et al., 2020). An important recent finding is a demonstration that the primate SC houses the first line brain neurons for automatic face detection (~50 ms after stimulus onset) (Le et al., 2020).

### The SC Role in Complex Innate Behaviors

Pivotal work on *sentinel SC functions* was undertaken by Merker in hamsters. Animals with SC lesions demonstrated severe deficits with respect to escape from moving visual threats (Merker, 1980). Several high-density electrode recording studies in optogenetically altered animals, as well as viral or molecular tracing studies, have demonstrated that the SC in rodents mediates a great deal of *innate* behavior, e.g., prey capture (Gahtan, 2005; Hoy et al., 2019), visual avoidance (Dong et al., 2009), and defensive escape, or freezing behaviors (Shang et al., 2015, 2018; Wei et al., 2015; Evans et al., 2018; Reinhard et al., 2019; Isa et al., 2020; Sans-Dublanc et al., 2021). These behaviors are triggered by complex images like body postures, body movements (e.g., attacking or escaping), facial expressions, or eye-related features. Additionally, they are influenced by environmental aspects as well as the emotional state of the animal (Sewards and Sewards, 2002; da Silva et al., 2013; Rosa Salva et al., 2015; Wei et al., 2015; Dunn et al., 2016; Huang et al., 2017; Ito et al., 2017; Liu et al., 2018; Shang et al., 2018; Lischinsky and Lin, 2019; Zhou et al., 2019; Daviu et al., 2020; Isa et al., 2020; Niu et al., 2020). The networks that mediate these reactions are segregated based on sensory input; as different retinal ganglion cell types record specific visual features of threatening images and activate diverse collicular networks (Gale and Murphy, 2014, 2018; Reinhard et al., 2019), and defined cell types in the SC make distinct contributions to prey capture behaviors (Hoy et al., 2019).

Sensorimotor innate reactions of the SC are further modulated by input from the parabigeminal nucleus (Huda et al., 2020), and from the prefrontal and anterior cingulate cortex (Huda et al., 2020; Tokuoka et al., 2020). The stimulation of different SC descending pathways induces contraversive head/body turns or defense-like behaviors. It is noteworthy that these responses can vary considerably depending on the environment where the animals were tested, so they are context-specific (Isa et al., 2020).

Connections between the substantia nigra (SN) and the SC may play a pivotal role in behaviors driven by *extrinsic motivation* in order to promote survival and reproduction (Comoli et al., 2003; May et al., 2009; Redgrave, 2010; Isa et al., 2020). They are also activated during *intrinsic motivation* triggered by novel stimuli and even by the pleasure of acquiring knowledge or new skills (Fisher et al., 2014; Caligiore et al., 2015). Highly segregated SC loops provoking either defense or approach behaviors also involve the cerebellum, the basal ganglia, and modulatory inputs from *cholinergic* (pedunculopontine nucleus and tegmental nucleus), *noradrenergic* (locus coeruleus), *dopaminergic* (retrorubral area), and *glutaminergic* nuclei (Redgrave, 2010; Comoli et al., 2012), as well as *serotoninergic* cells (raphe nucleus) (Huang et al., 2017). Phylogenetically, all these areas precede the expansion of the cerebral cortex by several 100 millions of years.

In rodents, it has also been shown that visual information from collicular superficial layers is highly *filtered* before reaching the deeper layers in order to develop an increased selectivity for the behaviorally relevant looming stimulus over other innocuous stimuli with similar low-level features; an increasing invariance to the precise location of the threat stimulus; and an increased selectivity for a novel over familiar stimuli (Lee et al., 2020). Additionally, looming images simulating flying predators, cause the SC to stimulate *corticotropin-releasing hormone* (CRH) neurons in the paraventricular nucleus of the hypothalamus, resulting in stress responses and defensive behavior in rodents (Daviu et al., 2020).

### Multisensory Integration, Motor Functions, and Orienting Attention: The Role of the SC

After the neonate begins to *experience* repeated multimodal events congruent in time and/or space (visual and auditory), *multisensory/pre-motor single neurons and complex multimodal networks start to appear in the intermediate SC layers*. This training occurs under the double influence of the environment and cortical regions (Alvarado et al., 2008; Stein et al., 2009; Bauer et al., 2012, 2015; Xu et al., 2014; Wang et al., 2020). The deeper SC layers receive whole body somatosensory input with a larger representation of the face. The alignment of visual and sensory maps within the SC layers has been proposed as the functional substrate to create the newborn's minimal intersubjective mind (Pitti et al., 2013). *An interaction between the SC, the PAG, and dopamine systems originating in the midbrain ventral tegmental area has been proposed as the fundamental neural substrate for complex intersubjectivity* (Corrigan and Christie-Sands, 2020).

In order to represent the environment coherently, the brain automatically creates *illusions* of reality. Some illusions are unisensory, while others result from the integration of two senses; for example: when a single flash is presented along with two or more beeps, observers often report seeing two or more flashes (fission illusion). The *McGurck effect* involves visual and auditory fusion (Mcgurk and Macdonald, 1976). *The Ventriloquist effect* apparently adds *semantic congruence* to multisensory/motor integration: we know from previous experience that a voice is produced by lip movements (Wallace et al., 2004; Ursino et al., 2014). Even when we explicitly know that the source of the sound is elsewhere, it is extremely difficult or even impossible to overcome the illusion the dummy speaks in order to find the source of the voice. Evidence indicates that multisensory neurons present on intermediate/deep SC layers play a key role in these phenomena (Stein et al., 2009, 2014; Ursino et al., 2014).

Connections between the SC and the cerebellum provide both the initial command, which generates a saccade and the error signal that ensures saccades remain accurate (Soetedjo et al., 2019). Evidence suggests that *these loops have not only motor but also purely cognitive, functions* which have been linked with ASD and other DD (Sathyanesan et al., 2019). Apparently, several SC brainstem, thalamic, and basal ganglia loops modulate emotional, sensory, and motor variables in order to make automatic decisions in saccade production (Caligiore et al., 2013; Thurat et al., 2015; Solié et al., 2022). Evidence indicates that following *Bayesian* instead of “winner takes all” rules, the motor reaction produced by interacting forces at the SC reflects a saccade choice instead of a simple saccade vector (Basso and May, 2017). Several multisensory, Bayesian, or Hebbian neurocomputational schemes have been proposed to explain the dynamics of the integration required to register the world coherently, and the SC is considered the crucial structure in all of them (see Ursino et al., 2014 for a review).

All these behaviors seem to fit under the concept of *embodied cognition* or “*enaction.” This concept* maintains that cognition and language emerge as a result of the active sensorimotor interaction between the agent and its environment (Heinrich et al., 2020), where movements guided by vision play a central role. It also includes the concept of “*autopoietic enactivism”* which means that through active environmental interactions the agent has the ability to self-individuate, self-regulate, self-develop, self-maintain, and reproduce (see Wilson and Foglia, 2017 for a review).

### Top-Down Direct Cortical Influence on the SC

Inside the cortex, neurons in all the cortical layers provide input to large pyramidal neurons in the 5th layer in each specialized region of the cerebrum. The descending axons of many of these layer 5 pyramidal neurons establish *direct* connections with the SC, rendering it functionally an additional cortical layer that is targeted by this cortical outflow pathway (Merker, 2013). For example, recently, it was demonstrated in mice that prefrontal cingulate corticotectal pyramidal neurons enhance saccade planning and visual processing through projections to the SC (Hu et al., 2019).

We can conclude that the SC combines first-line environmental and efference copy signals with the body (from peripheral somatosensory input) and cortical information. In this way, a resonance between the implicit bottom-up reality created at the SC is amalgamated with the colorful, detailed, and dynamic multisensory conscious reality created by higher-order cortical loops. Ultimately, this integration allows a continuous “global best estimate” of sensory, motivational, and motor circumstances in order to act coherently and effectively (Merker, 2013).

### Embodied Cognition, the *Self*, and the SC

Dynamic embodiment theories are mainly based on complex *visual stability* achieved by the individual in their environment (Wilson and Foglia, 2017). The SC's integrative analysis of first-line environmental, motor, and higher-order information seems to fulfill the functional requirements to achieve this goal. A thoughtful description of SC functions and its interplay with cortical activity to produce the emergence of visual consciousness under the perspective of the *self* is given by Merker (2013).

The embodied concept also applies to mental abilities. Current evidence suggests that we understand others' emotions and intentions by recreating, at least partially, sensorimotor experiences in our own bodies. In spite of the current consensus and evidence supporting embodied theories, as opposed to body/mind dualism, there is still a debate about the degree of embodiment (Ursino et al., 2014; Wilson and Foglia, 2017). Independent of which neurocomputational scheme is proposed, the SC is considered a key structure for explaining the integration required to register the world coherently (Ursino et al., 2014). It functions as the kernel between the bottom-up input from the body and the environment and the top-down influence of cortical functions.

### Plastic and Cognitive Influence of the SC on Normal and Abnormal Neurodevelopment: Neurophysiological Evidence

Animal studies have shown that in early life the SC fosters the creation of Pul-cortical loops that support visual cognition (Bridge et al., 2015). Unlike the dLGN, which matures later (Sewards and Sewards, 2002), the SC is not dependent on postnatal input from cortical areas as it can accomplish *visual sensory functions* in decorticated mice (Shanks et al., 2016). Furthermore, it plays a fundamental role in supporting the neuronal differentiation induced in other structures by retinal input (Alvarado et al., 2021). Through interactions between cell surface molecules and their ligands, the SC develops a series of topographically organized connections (Scicolone et al., 2009; Mendonça et al., 2010; Triplett and Feldheim, 2012; Carr et al., 2013; Chagas et al., 2019). Specific SC mechanisms promote topographic cortical alignment of visuals with somatic input (Triplett et al., 2012). It also acts as a *driver* or *modulator* providing complex motion information to the dLGN, in order to integrate convergent information about stimulus motion, eye movement, and positioning in the visual primary cortex (Bickford et al., 2015).

### Summary of SC and Brainstem Functions

The SC and its brainstem loops concentrate several functions necessary to produce behaviors allowing most vertebrates to survive and reproduce. The preservation of this machinery from early vertebrates to humans bespeaks its great efficacy and its irreplaceable character. These functions, triggered mainly by postnatal biological visual input, allow not only for defense, attack, or escaping behaviors, but they also give every creature the sense of self, the ability to differentiate inanimate objects from living beings, to differentiate familiar people from strangers, to respect hierarchies, and to create Bayesian patterns in order to make the right decision at the right time. The lack of *simultaneous* evaluation of several variables of the environment, the self, and other creatures' intentions in order to produce a fairly automatic action with the correct timing is incompatible with self-preservation. This low-level input-output processing is mainly automatic. Perceptual, emotional, and motor aspects cannot be fully separated due to their interdependence. Highly suggestive evidence for this holistic processing is the appearance, very early in life, of individual multisensory/premotor SC neurons with visual, auditory, somatosensory, autonomic, and emotional functions that produce an automatic motor output along with an attendant shift in attention (Meredith et al., 1992; Stein et al., 2009). Accordingly, each action or behavior is preceded by perception and emotions, and a perfect balance between different emotions is necessary in order to make the right decision (for example the mother that, overcoming her own fear, decides to attack a dangerous animal to defend her offspring). We can conclude that the SC and its surrounding structures *are not only responsible for the initial visual bias to global perception, but they also create holistic patterns that involve multisensory-emotional, autonomic, and motor aspects*. As it is difficult to separate between the perceptual, emotional, attentional, and motor theories of autism, it is also difficult to separate the functional confluence of these aspects in the SC.

## Correlations Between Asd And Sc Disruptions

**Figure 1:**
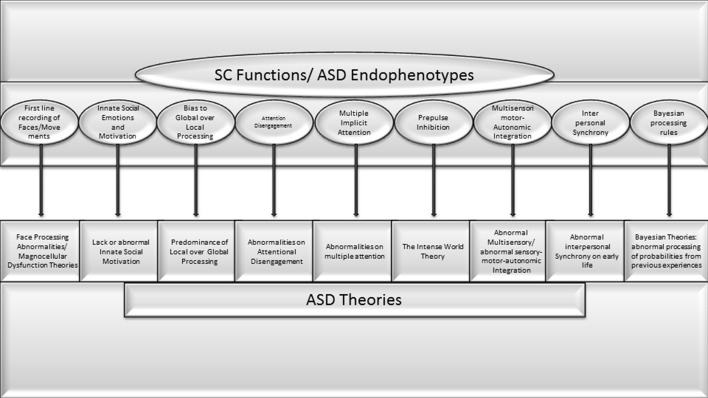
Very high concorndance between SC functions and endophenotypes or pivotal symptoms of ASD theories previously proposed.

The comparison of previous theories, pathogenic and etiologic aspects of ASD with SC functions demonstrate a high level of coincidence (See Figure 1). Some of the following points are pivotal aspects of ASD that depend on brain functions that, according to clear scientific evidence, are exclusively performed by the SC. Other symptoms depend on functions in which the SC plays a prominent role, but it is shared by other brain structures. For others, strong evidence of exclusivity is still lacking, but a possible role in development is suspected:

- *The atypical automatic bias for global instead local visual processing is the cornerstone of several prominent autism theories* (Van der Hallen et al., 2015). *The superficial visual SC is the exclusive structure responsible for this initial automatic bias to global processing* (Lomber, 2002; Kato et al., 2011).
- *Numerous ASD theories are based on abnormal processing of faces and movements, and abnormal innate social motivation. The SC houses the first line neurons for automatic face recognition* (Le et al., 2020). The lack of responses to faces and biological movement is present from birth in a significant proportion of individuals with ASD (NIDA-Network et al., 2016).
- A very primitive animal behavior is the automatic *response to looming images* representing moving visual threats. In mammals, this function depends *exclusively on input-output processing for SC sentinel functions* (Merker, 1980). *The lack of innate looming-evoked defensive response observed in a significant proportion of children with ASD* and in mice prenatally exposed to valproic acid has been attributed to a dysfunction in the subcortical pathway involving the SC (Hu et al., 2017).^2^
- *Retinocollicular projections* also deserve special attention because *a compromise of these fibers* could be secondary to different nutritional (including a chronic restriction of omega-3 fatty acids), toxic, infectious, hormonal factors, or microglial activation during key developmental periods *resulting in ASD* (Chagas et al., 2020; Sandre et al., 2021).
- Interrupting an ongoing behavior is also essential for social interaction. Several authors have proposed that *abnormal disengagement of visual attention during infancy is the underlying abnormality in ASD* (see above). A specific *subpopulation of disengagement SC neurons* has been described in rodents by Ngan et al. (2015).
- Abnormal response to sensory stimuli and abnormal pre-pulse inhibition (PPI) response were proposed in the *intense world theory* as one of the markers of ASD (see above). Dendrinos et al. (2011) *specifically* linked the compromise of SC parvalbumin containing GABAergic neurons to impaired PPI in rodents prenatally exposed to VPA. More recently, the critical role of the SC was demonstrated in macaques by a frank compromise of PPI to acoustic startle response after bilateral inhibition of collicular deep/intermediate layers (Waguespack et al., 2020).
- *The SC is the main brain hub that integrates sensory, motor, emotional, and autonomic dimensions*. A variety of ASD theories are based on abnormal multisensory functions (Stevenson et al., 2014; Camarata et al., 2020; Kawakami et al., 2020; Siemann et al., 2020). Motor, autonomic, emotional, and several other variables are included in *the micro-movement perspective theory* (Torres et al., 2013) and the *abnormal interpersonal synchrony theory* (McNaughton and Redcay, 2020). Similarly, *Bayesian theories of ASD* are based on patterns of multisensory integration (French and DeAngelis, 2020). The abnormal synergic activity of multisensory SC neurons with a cascade effect on neurodevelopment has been linked with ASD and dyslexia (Siemann et al., 2020; Wang et al., 2020). Several multisensory, Bayesian, or Hebbian neurocomputational schemes have been proposed to explain the dynamics of the integration required to register the world coherently, and *the SC is considered the integrative hub for all these variables* (see Ursino et al., 2014 for a review).
- The *weak central coherence theory attributes ASD to a reduction in synchronization of high-frequency gamma activity* (Brock et al., 2002). Gamma band abnormalities have been found in individuals with autism during perceptual tasks (Brown et al., 2005), gaze cueing (Richard et al., 2013), and emotional face processing (Safar et al., 2020). *Gamma band synchronization only appears in cortical, SC, and pulvinar regions* (Fries, 2009; Bastos et al., 2015; Bryant et al., 2015; Baranauskas et al., 2016; Le et al., 2019). It is also associated with *cholinergic* (Bryant et al., 2015) and *GABAergic activity* at the SC (Nakamura et al., 2015).
- Postnatal environmental input is essential to activate parvalbumin-positive GABAergic neurons present in different subcortical areas. These neurons orchestrate brain organization promoting an inhibitory-excitatory balance (Takesian and Hensch, 2013). The SC has a high density of GABA, with plastic functions during the perinatal period, and GABA acts as a “pioneer neurotransmitter” responsible for environment-induced synaptic architecture (Grantyn et al., 2011). Early alterations of GABA and glutamate may result in ASD and other DD (Grantyn et al., 2011; Horder et al., 2018; Li et al., 2018b). In fact*, GABA dysfunctions have been proposed as a biomarker for ASD* (Maxwell et al., 2015), *and reduced numbers of GABAergic SC neurons have been associated with autistic-like behavior in knockout mice* (Nakamura et al., 2015).
- The SC contains several subpopulations of parvalbumin-positive, GABAergic neurons, some of which are exclusive inhibitory; but unlike the cortex, it contains *subpopulations of parvalbumin-positive cells with glutamatergic excitatory activity* (Villalobos et al., 2018). Inhibitory (GABAergic) and excitatory (glutamatergic) activities have an influence on the neuroligins responsible for the postnatal postsynaptic balance between AMPA (Ca^++^ excitatory) and GABA (Cl^−^ inhibitory) receptors (Johnston and Blue, 2006). Numerous (~30) genetic mutations and pathogenic pathways affecting these neuroligins, GABA and AMPA have been linked with ASD (Johnston and Blue, 2006; Trobiani et al., 2020). In animals, *prenatal VPA exposure selectively affects parvalbumin-positive GABAergic neurons of the SC, provoking autistic-like symptomatology* (Dendrinos et al., 2011; Wöhr et al., 2015).
- The *interplay of GABA and serotonin* between the SC and dorsal raphe nucleus translate threatening looming visual signals into defensive responses (Huang et al., 2017), and both neurotransmitters have been linked with ASD pathogenesis (Skuse, 2006; Robertson et al., 2016; Di et al., 2020; Carvajal-Oliveros and Campusano, 2021).
- Other molecules linked with specific behavioral innate approach, defensive, or attack responses triggered by the SC and related to ASD pathogenesis are *acetylcholine* (Tokuoka et al., 2020), *endogenous opioids* (da Silva et al., 2013), *dopamine* (Redgrave, 2010; Solié et al., 2022), *corticotropin-release hormone* (Daviu et al., 2020), *adrenergic SC receptors and norepinephrine* (Iigaya et al., 2012; Li et al., 2018a; London, 2018), and *glutamine* (Barbano et al., 2020).
- *Nitric Oxide* is a key molecule in plastic developing pre-and postnatal periods expressed in the SC (Scheiner et al., 2001; Giraldi-Guimarães et al., 2004) and it is especially linked with Shank3 mutation and ASD (Tripathi et al., 2020).
- Very recently, *the neurexin gene family* has been implicated in ASD and apparently linked with post-synaptic changes. It was found to be *differentially* expressed within specific populations in the larval tectum. *This strongly suggests a potential genetic link between the SC/tectum and ASD* (Martin et al., 2022).
- Lastly, but critically, is the *timing of occurrence of brain changes*. It must precede clinical manifestation within a circumscribed age window from prenatal to early post-natal when subcortical centers shape several brain regions through ascending connections. Synaptic postnatal growth reaches its maximum at 12 months and is followed by a massive pruning during early childhood (Vértes and Bullmore, 2015). An alteration in this balance might be responsible for the early brain overgrowth frequently observed in children with autism (Courchesne et al., 2007), as well as the abnormalities in “growth connectomics”—the organization and reorganization of brain networks during development (Vértes and Bullmore, 2015). Unlike the LGN, the SC is ready at birth to accomplish complex visual functions (Sewards and Sewards, 2002), and likely plays a primary role in the development of cerebral organization during this age window.

## Discussion

As was previously mentioned, the lack of association of total congenital deafness and acquired blindness with ASD contrasts sharply with the very high prevalence of ASD in CB. Even children with peripheral (i.e., non-neurological) etiologies of visual impairment that improved from profound to less severe vision compromise after the first year of life show a higher risk of autism regression later (between 15 months and 3 years of age) (Dale and Sonksen, 2002). Additionally, a very high prevalence of ASD has been described in *orphan children with severe environmental deprivation* during the first 6 months of age (Green et al., 2016). The main variable that might explain this difference is the complete lack of early exposition of visual experience, especially faces, human movements, and non-verbal aspects of interaction and communication. Similarly, *a crucial aspect of the present theory is that, in order to result in ASD, SC dysfunction should be congenital or acquired very early on, during the first months of life*.

It is also worth noting that given all SC connections, the existence of an *exclusive* compromise of its inner structure without an impact on several brainstem and thalamo-cortical structures is not possible. Similarly, it is not possible to compromise any neurotransmitter (GABA, serotonin, etc.) or gene responsible for synaptic changes exclusively in the SC without affecting other structures. However, selective compromise of the retino-colliculi-thalamic or the retino-geniculate pathway is possible due to their relative independence. Considering, hypothetically, that the first network fosters the development of higher-order behaviors dependent on primitive brain functions (including, for example, mental abilities), and the second the higher-order pathway functions *mostly* independent of primitive behaviors, such as logic, reasoning skills, or motor skills that are exclusively human, such as hand dependent abilities. Then, the selective dysfunction of these networks, or the compromise of the interaction between them, might have a great variety or spectrum of cognitive profiles distributed in a continuum, from normal to abnormal behaviors or abilities. These would in turn explain the broad ASD spectrum of clinical manifestations and its superposition with typical individuals in the less affected group.

It is not possible in this discussion to cover all the varieties of ASD symptoms, but the author invites clinicians working with individuals with ASD to use this framework to understand not only the classic symptoms but the paradoxes and the full range of the clinical spectrum, including comorbidities. For example, against the traditional view that oral language emerges first and then fosters neurodevelopment, evidence indicates the contrary. In human babies, the ability to retain and later recreate a sequence of movements is the base of representational play, which emerges shortly before language, with a close correlation between the complexity of representational play and the complexity of language production (McCune and Zlatev, 2015). Simple or deictic communicative gestures like pointing appear near 12 months. These behaviors require motor control and directed attention for triadic interactions (child, object, and other people) for normal function, as well as social/cultural learning, and the understanding that other persons have thoughts and intentions (McCune and Zlatev, 2015). This denotes the importance of early visual attention to social clues and body movements.

Independent of the frequent comorbidity with cognitive deficits and different developmental language disorders (DLD) (Rapin and Allen, 1983; Rapin and Dunn, 2003) abnormalities in these pre-requisites for language development explain several atypical findings, mostly observed in individuals with ASD, and not with DLD without ASD. For example, total attentional neglect to communication and language during the first year of life will result in children obtaining things for themselves or by taking the hand of a caretaker instead of pointing or naming it to get what they want. Subsequently, the lack of discrimination between self and non-self (and its language correlate) could result in echolalic, third person, or rote memory expressions about specific topics like TV commercials, songs, the alphabet, numbers, geometric figures, etc. Patients with ASD monolog about these topics, instead of sharing, in a triadic interaction, a topic of mutual interest. The presence of difficulties referring to events distant in time or space, or problems understanding and expressing narratives (Paolucci, 2020) could be explained by the lack of imaginative play (which is mainly based on images). All this evidence indicates the importance of basic sensorimotor, as well as emotional dimensions, in the development of verbal and non-verbal communication. They also support the theories pointing to embodied language and cognition (see above).

Additionally, SC abnormalities affecting sentinel and integrative sensory-motor and emotional variables might explain abnormalities in automatic reaction to an injury (e.g., a burn) or any dangerous situation that requires innate, primitive reactions (escaping, defense, freezing, etc.). The lack of normal filtering of information by the SC in order to lessen the load of irrelevant detail for the rest of the brain might explain a number of ASD symptoms, such as the abnormal response to sensory stimuli and the great memory for details.

Abnormalities in first-line, automatic, holistic multisensory, autonomic, emotional, and motor representations supported by the SC might affect the creation of Bayesian patterns after repeated expositions to similar events. This dysfunction might explain the difficulty in individuals with ASD to generalize and learn from experience in order to react automatically with the right timing. Also, as the colliculus automatically combines multiple attentional sources, including own-body sensorimotor perception, several external clues, and numerous cortical inputs, it integrates body-mind-environment information. This seems necessary to manage multiple clues and think appropriately about them, as well as to understand one's own body or emotional feelings. Many with ASD suffer deficits in these areas.

As was remarked upon earlier, the coexistence of autistic symptoms with normal or high intelligence in a significant proportion of the spectrum might be explained by the compromise of the primitive brain without affecting higher-order cerebral functions. Developmental motor abnormalities–mainly dyspraxia–frequently observed in ASD individuals could be the result of a different way of motor learning. This learning is more conscious and focal, which explains the presence of disparities, not only in cognitive or sensory areas but also in motor performance.

An improper feedforward and feedback interaction between the primitive brain and higher structures beginning early in life might result in a lack of balance, with the consequent development of hyperactive subcortical circuits. This, in turn, could explain the abnormalities in emotional reactions, excessive anxiety, stereotypies, and obsessive thinking among the symptoms present in ASD.

Several of the neurotransmitters previously mentioned also play a central role in the pathogenesis of other DD. These might explain the ubiquitous presence of one or more comorbidities such as ADHD, learning disabilities, sleep and mood disorders, anxiety, obsessive-compulsive disorders, epilepsy, cognitive deficits, and autonomic abnormalities that are all found in individuals with ASD (Casanova et al., 2020).

## Final Conclusion

Compromise of the SC in ASD was previously proposed by the present author (Jure, 2019). The present publication adds new evidence supporting this hypothesis. It also makes clear the importance of the SC for numerous brainstem structures and loops present in the primitive brain. If there is early collicular dysfunction, this network can act as a bottleneck in the development of social-communicative abilities. Additionally, I have suggested here a new holistic framework of initial bottom-up collicular processing.

An early dysfunction of the primitive brain offers the most unifying theory of ASD pathogenesis. The SC, as the main brainstem hub, accomplishes several primitive functions whose compromise explains not only the core and the accompanying symptoms of ASD, including their usual presentation in clusters, but also the presence of a clinical spectrum, replete with uneven skills, and comorbidities.

Instead of the *exclusively visual* “global” vs. “local” dichotomy, a new framework of information processing is suggested here. The new proposal is that the SC and the networks it activates have a holistic registration of external events that includes multisensory/motor/emotional/autonomic aspects that occur simultaneously and automatically. The SC also helps filter out unnecessary or superfluous details. This kind of processing gives several benefits that are frequently abnormal or absent in ASD individuals.

Disruptions of SC functions may provide the neurologic substrate that encompasses *all* previous theories. Genetic and/or non-genetic prenatal and postnatal etiologic and pathogenic factors may affect the SC and lead to ASD because it is a primary structure for fostering brain plastic changes by epigenetic influence during early postnatal life. This extremely active period shapes the future individual regarding both basic sensory/motor/autonomic aspects, as well as cognitive, social, and emotional higher-order abilities. For this reason, numerous neurotransmitters and neurotrophic and signaling molecules are likely to play a role during this age window, which coincides with the initiation of autistic symptoms. Dysfunction in any of these may play a crucial role in this initiation. This also fits with the findings of genetic and non-genetic compromise of retino-collicular axon guidance formation as pathogenic factors for ASD (Campello-Costa et al., 2000; Lee et al., 2010; Brielmaier et al., 2012; Chagas et al., 2019, 2020). The elusive pathogenesis of ASD, which has not been determined even after decades of research on genes and cortical structures, could lie in the epigenetic and plastic effects that the SC and related brainstem nuclei provoke all over the brain during early life.

To test this theory, future studies need to focus on post-mortem SC observation or non-invasive functional human studies of the colliculus and its targets (e.g., the pulvinar), as well as animal studies undertaken during the critical collicular-driven developmental period. For example, studies regarding the early post-natal activation of subpopulations of SC neurons by specific genes involved in both the development of the SC and the pathogenesis of ASD would be helpful. Developmental changes in subpopulations of GABAergic neurons directly implicated in ASD animal models should also be targeted. It might be useful to develop animal models of ASD in species that show higher levels of social interaction and verbal communication (e.g., chinchillas). Finally, larger prospective studies regarding the development of subtle aspects of social and verbal or non-verbal communicative abilities in individuals with congenital *vs*. acquired blindness are needed.

The references in the present article represent only a small fraction of the indirect evidence of literature supporting the relationship between the SC and different aspects of ASD. Additionally, the SC is only one of the many brainstem nuclei with an influence on neurodevelopment. All of them are highly interconnected. It is possible that the knowledge of their influence on normal and abnormal human development will require new techniques and decades of investigation. Nevertheless, this knowledge might help us to develop more effective therapeutic or preventative tools.

## Data Availability Statement

The original contributions presented in the study are included in the article/supplementary material, further inquiries can be directed to the corresponding author.

## Author Contributions

RJ a child neurologist specialized in neurodevelopmental disorders, provides a Unifying Theory of Autism based on a review of the literature showing new evidence about the role of primitive structures such as the superior colliculus and brainstem related structures on early postnatal brain development.

## Conflict of Interest

The author declares that the research was conducted in the absence of any commercial or financial relationships that could be construed as a potential conflict of interest.

## Publisher's Note

All claims expressed in this article are solely those of the authors and do not necessarily represent those of their affiliated organizations, or those of the publisher, the editors and the reviewers. Any product that may be evaluated in this article, or claim that may be made by its manufacturer, is not guaranteed or endorsed by the publisher.

## Footnotes

^1^Experience-expectant refers to the fact that the average or normal environment provides infants with the necessary input to develop the neural connections to enable the baby to function across these domains.

^2^A selective compromise with uneven cognitive skills was also observed in one of my patients with ASD secondary to fetal valproate syndrome. He/she spoke fluently with good verbal and visual memory, but displayed severe deficits in socialization, communication and pragmatic abilities. This finding suggests that the compromise of synapses by prenatal valproate exposure is not ubiquitous, but is instead selective and might be related with autistic symptoms dependent on specific SC functions.
